# A Proline-Rich Element in the Type III Secretion Protein FlhB Contributes to Flagellar Biogenesis in the Beta- and Gamma-Proteobacteria

**DOI:** 10.3389/fmicb.2020.564161

**Published:** 2020-12-15

**Authors:** John C. Hook, Vitan Blagotinsek, Jan Pané-Farré, Devid Mrusek, Florian Altegoer, Anita Dornes, Meike Schwan, Lukas Schier, Kai M. Thormann, Gert Bange

**Affiliations:** ^1^Department of Microbiology and Molecular Biology, Justus-Liebig-Universität, Giessen, Germany; ^2^SYNMIKRO Research Center, Department of Chemistry, Philipps-University Marburg, Marburg, Germany

**Keywords:** flagellum, flagellum assembly, crystal structure, FlhB, filament, bacteria, microbiology

## Abstract

Flagella are bacterial organelles of locomotion. Their biogenesis is highly coordinated in time and space and relies on a specialized flagellar type III secretion system (fT3SS) required for the assembly of the extracellular hook, rod, and filament parts of this complex motor device. The fT3SS protein FlhB switches secretion substrate specificity once the growing hook reaches its determined length. Here we present the crystal structure of the cytoplasmic domain of the transmembrane protein FlhB. The structure visualizes a so-far unseen proline-rich region (PRR) at the very C-terminus of the protein. Strains lacking the PRR show a decrease in flagellation as determined by hook- and filament staining, indicating a role of the PRR during assembly of the hook and filament structures. Phylogenetic analysis shows that the PRR is a primary feature of FlhB proteins of flagellated beta- and gamma-proteobacteria. Taken together, our study adds another layer of complexity and organismic diversity to the process of flagella biogenesis.

## Introduction

The flagellum is a highly conserved structure used by bacteria to move through liquid solutions and over surfaces. The overall architecture of the flagellum can be divided into the membrane-embedded basal body, the rod, the extracellular hook, and filament (summarized in: Chevance and Hughes, 2008; Nakamura and Minamino, 2019). The biogenesis of the flagellum is highly orchestrated in time and space and relies on a specialized flagellar type III secretion system (fT3SS) localizing at the center of the basal body (Macnab, 2003; Altegoer and Bange, 2015). The fT3SS consists of the transmembrane proteins FlhA, FlhB, FliOPQR, and the soluble ATPase FliI together with its regulators FliJ and FliH (summarized in: Macnab, 2004). The fT3SS is distinct from injectisome-related type III secretion systems found in pathogenic organisms that use it to secrete toxins and virulence factors into host cells. While both systems differ on a functional and physiological level, the components of their T3S apparatus are highly similar at the level of sequence and structure (Macnab, 2004; Erhardt et al., 2010; Abrusci et al., 2014). However, at the extracellular side, the injectisome contains a needle for injection of virulence proteins into the host cell, while the flagellum is characterized by the rod, hook, and filament structures.

**FIGURE 1:**
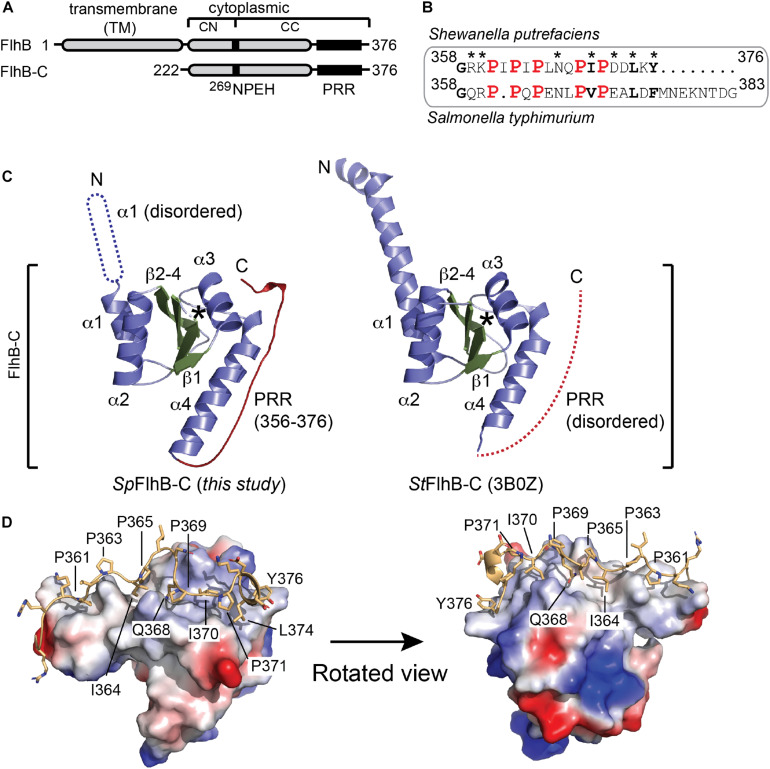
Structural analysis of *Sp*FlhB-C. **(A)** Domain architecture of FlhB and the FlhB-C variant used for structural analysis. **(B)** Sequence alignments of *S. putrefaciens* and *S. typhimurium C-termini*. **(C)** Crystal structures of FlhB-C from *S. putrefaciens* (*Sp*FlhB-C; left, this study) and *S. typhimurium* (*St*FlhB-C; right, 3B0Z). The β-strands 1–4, α-helices 1–4, and the PRR are shown in green, blue, and red, respectively. Protein regions not visualized in the structures are indicated as dotted graphical elements. Asterisks indicate the auto cleavage site. ‘N’ and ‘C’ indicate N- and C-termini, respectively. **(D)** The PRR exhibits extensive contacts to the core domain of *Sp*FlhB-C. The FlhB-C core domain is shown as electrostatic surface area. The PRR is shown as yellow cartoon with side chains. The prolines 361, 363, 365, 369, and 371 and side chains mediating interactions between PRR and core domain are indicated.

FlhB (also: YscU, SpaS, SctU, and Spa40 in the injectisome-related T3SSs; see also: Abrusci et al., 2014) was identified and initially characterized together with FlhA and FlhE in *Salmonella enterica* (Minamino et al., 1994). It was shown that FlhB consists of two regions—an N-terminal transmembrane domain (TMD) followed by a cytoplasmic domain (FlhB-C) (see Figure 1A). Recent structural analysis showed that the TMD of FlhB can be part of the FliPQR complex and as such is involved in the gating of the T3SS (Kuhlen et al., 2020). FlhB-C has the ability for self-cleavage between the asparagine and the proline of the conserved NP(T/E)H motif (Minamino and Macnab, 2000; Ferris et al., 2005). Autocleavage of FlhB-C results in two tightly associated subdomains, named FlhB-CN and FlhB-CC, located N-terminally and C-terminally to the cleavage site, respectively (Figure 1A) (Ferris et al., 2005; Zarivach et al., 2008). In *S. enterica*, mutating N269 and P270 completely inhibits and reduces the autocleavage, respectively. An alanine mutation of N269 abrogates formation of the flagellar filament due to defects in flagellin secretion, while an equivalent mutation of P270 yielded reduced flagellin export. Both mutants show the “polyhook” phenotype, which is characterized by significantly extended hook structures and the absence of a filament (Fraser et al., 2003). This polyhook phenotype caused by the FlhB mutations is highly similar to a deletion of the gene encoding FliK (Minamino et al., 1994; Williams et al., 1996; Meshcheryakov et al., 2013a, b). Further experiments showed a direct interaction of the FlhB and FliK proteins (Morris et al., 2010). Moreover, the deletion of a proline-rich region (PRR) at the very C-terminus of FlhB (CCT in: Kutsukake et al., 1994; PRR from here onward) was shown to result in the formation of filaments on top of the polyhook structure in the context of a *fliK* deletion (Kutsukake et al., 1994). Overall, these findings show that FlhB and FliK are important for the switching of the export specificity from rod- and hook-type substrates to filament-type substrates during flagellar morphogenesis (Williams et al., 1996; Minamino and Macnab, 1999; Minamino et al., 1999).

In this study, we have investigated the crystal structure of the FlhB protein (Sputcn32_2563) from the polar flagellum of the Gram-negative bacterium *Shewanella putrefaciens CN-32* (Bubendorfer et al., 2012, 2014). The bacterium is a monotrichous polar flagellate also capable of producing additional lateral flagella in high-nutrient and viscous environments. The lateral flagellar system contains individual copies of proteins, including FlhB, from its own distinct flagellar cluster (Sput32_3447-3485) (Bubendorfer et al., 2012). The number and localization of the polar flagellum is tightly controlled by the FlhF/FlhG pair, which are not known to interact with the lateral flagellum (summarized in: Schuhmacher et al., 2015). Our structural analysis visualizes a PRR at the very C-terminus of FlhB-C of the polar fT3SS. Our functional and phylogenetic analysis shows that this PRR is an exclusive feature of the gamma- and proteobacteria and contributes to the efficiency of hook and filament assembly, however does not impact C-ring assembly.

## Results

### Structural Analysis of FlhB-C Reveals a Proline-Rich Motif (PRR) at Its C-Terminus

First, we wished to determine the crystal structure of the cytoplasmic domain of FlhB of the main polar flagellar system [FlhB_1_ (Sputcn32_2563), henceforth simply FlhB], which localizes at the C-terminus of the protein (*Sp*FlhB-C; amino acid residues: 222–376) (Figure 1A). This FlhB variant was purified by a two-step protocol consisting of a Ni-ion affinity- followed by size exclusion chromatography (SEC). As for other FlhB proteins, the *S. putrefaciens* FlhB also undergoes autocleavage at the conserved ^269^NPEH motif resulting in the FlhB-CN and FlhB-CC fragments. An alanine variation of the catalytically relevant Asn269 completely abolishes the autocleavage of FlhB-C. Visualization of the WT and N269A FlhB by means of a Western blot is discussed in more detail later on, where the cleavage is also quantified (see below).

The crystal structure of FlhB-C was determined by molecular replacement employing the structure of *Salmonella typhimurium* FlhB-C as search model, and refined to a resolution of 2.1 Å (Table 1). An alignment of the *S. putrefaciens* and *S. typhimurium* FlhB C-termini (PRR regions) showed that all five proline residues in the PRR are conserved (Figure 1B). The final model comprises residues 252–376 of *Sp*FlhB-C and compares well with the previously determined homologous structures from *S. typhimurium* and *Aquifex aeolicus* [PDB-IDs: (3B0Z) and (3B1S), respectively; Meshcheryakov et al., 2013b). The root mean square deviations (r.m.s.d.s) between the here presented structure of *Sp*FlhB-C and those of *S. typhimurium* and *A. aeolicus* are 0.892 and 0.913 over 519 Cα-atoms, respectively, further supporting the high structural similarity among FlhB-C domains. All FlhB-C structures share the central domain composed of a four-stranded β-sheet in its center, which is surrounded by four α-helices (Figure 1C).

**TABLE 1:** Data collection and refinement statistics.

	*Sp*FlhB-C
**Data collection**	
Space group	R 3 2
**Cell dimensions**	
*a*, *b*, *c* (Å)	152.436 152.436 126.886
α, β, γ (°)	90, 90, 120
Resolution (Å)	46.44 − 2.1 (2.175 − 2.1)
*R*_merge_	0.1412 (1.233)
*I/*σ*I*	13.46 (1.64)
Completeness (%)	99.96 (99.94)
Redundancy	19.9 (18.3)
**Refinement**	
Resolution (Å)	46.44 − 2.1
No. reflections	33095 (3286)
*R*_work_/*R*_free_	0.21/0.24
No. atoms	4134
Protein	3932
Ligand/ion	–
Water	202
*B*-factors	48.30
Protein	48.31
Ligand/ion	-
Water	47.97
Ramachandran favored (%)	97.88
Ramachandran allowed (%)	2.12
Ramachandran outlier (%)	0.00
**R.m.s. deviations**	
Bond lengths (Å)	0.011
Bond angles (°)	1.16

*Values in parentheses are for highest-resolution shell.*

As observed for all other FlhB-Cs analyzed so far, autocleavage was also seen in our structure (Figure 1C). This autocleavage occurred at the highly conserved ^269^NPEH^272^ motif and leads to two tightly associated subdomains FlhB-CN and FlhB-CC (residues 252–269 and 270–376, respectively). However, the structure of *Sp*FlhB-C differs from the other available ones in two main features (Figure 1C): First, the N-terminal fraction of helix α1, which connects FlhB-C to its TM domain, appears disordered in our structure. Second, our structure of *Sp*FlhB-C resolves a PRR involving residues 356–376 at the very C-terminus of FlhB-C, which was not observed in previous structures. The PRR follows helix α4 of FlhB-C and appears as a “wave”-shaped linear motif (Figure 1D). The PRR interacts with an extended canyon-like surface area formed by helix α4 of FlhB-C. At its C-terminus, the PRR is also in close proximity to helix α3. The interface area between the core domain of FlhB-C and the PRR is 855 Å^2^ of buried surface area. Our analysis identifies the following hydrogen-bonding pairs between the PRR and helix 4 of the core domain: K360-Y353, G358-Q349, G358-Q352, R359-Q349, R357-Q352, Q368-Q342, and Q368-A339 (Supplementary Figure S1). Moreover, a number of additional residues of the PRR and helices 3 and 4 are involved in the formation of hydrophobic contacts (e.g., I364, I370, L374, and Y376) (Figure 1D and Supplementary Figure S1). Taken together, our structural analysis of *Sp*FlhB-C shows the high degree of structural conservation between FlhB-C domains. In addition, we could resolve the so-far structurally uncharacterized PRR at the very C-terminus of *Sp*FlhB-C, which binds into a well-defined surface area defined by α-helices 3 and 4 of the FlhB core domain (Figure 1D and Supplementary Figure S1).

### Removal of the FlhB PRR and Substitution of the Cleavage Site N269 Affect Both the Flagellar Hook Formation and Filament Assembly and Length

**FIGURE 2:**
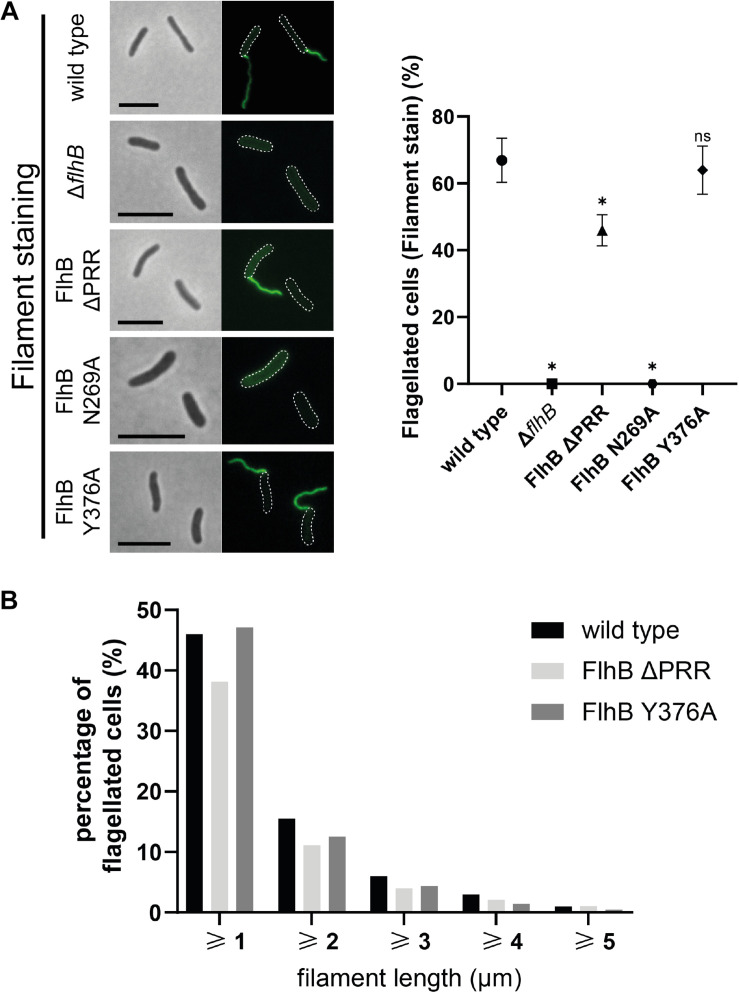
Flagellar filament microscopy, quantification, and length analysis of *Sp*FlhB ΔPRR, N269A, and Y376A effects in comparison to wild type and a Δ*flhB* mutant. **(A)** Microscopy images of filament stained cells and comparative quantification of filament formation in FlhB mutant strains with appropriate controls. **(B)** Comparative flagellar filament length analysis of wild type, *Sp*FlhB ΔPRR and Y376A. Scale bar: 5 μm. Statistical analysis was conducted via unpaired *t*-test for each mutant in comparison to the wild type. * =̂ *P*-value ≤ 0.0001.

**FIGURE 3:**
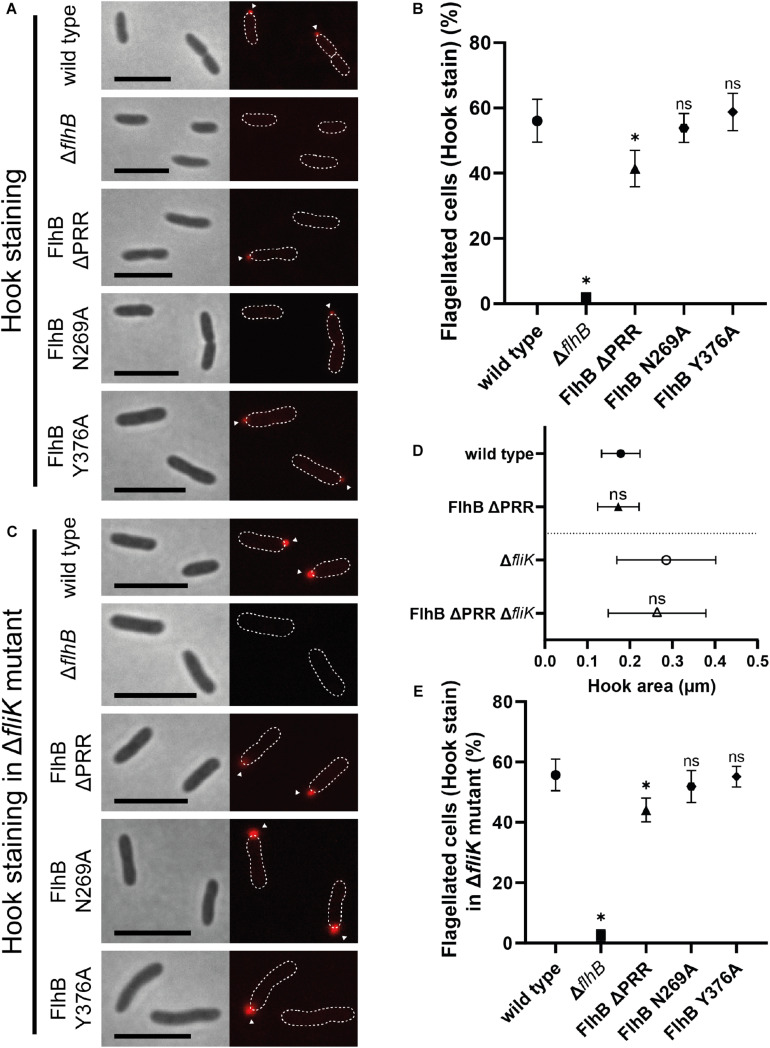
Flagellar hook microscopy and quantification analysis of *Sp*FlhB ΔPRR, N269A, and Y376A effects in comparison to wild type and a Δ*flhB* mutant in both a wild type and a Δ*fliK* background with additional hook area analysis of *Sp*FlhB ΔPRR and *Sp*FlhB ΔPRR Δ*fliK* in comparison to wild type and Δ*fliK*. **(A,B)** Microscopy images of hook stained cells and comparative quantification of hook formation in FlhB mutant strains with appropriate controls in a wild type background. **(D)** Comparative measurement of hook area of FlhB ΔPRR and FlhB ΔPRR Δ*fliK* strain in relation to the appropriate “wild type” (wild type and Δ*fliK*). Significances have been calculated relative to the appropriate “wild type.” **(C,E)** Microscopy images of hook stained cells and comparative quantification of hook formation in a Δ*fliK* background. Scale bar: 5 μm. Statistical analysis was conducted via unpaired *t*-test for each mutant in comparison to the wild type. * =̂ *P*-value ≤ 0.0001.

Our structural analysis visualized the PRR present at the very C-terminus of FlhB, which interacts with the core domain of FlhB-C. In order to gain a better understanding of the functional relevance of the PRR for flagellar assembly, we constructed *S. putrefaciens* strains in which either the PRR or complete *flhB* were deleted and either the cleavage site N269 or the Y376, localizing at the C-terminal end of the PRR and providing hydrophobic contacts to the FlhB-C core domain, were substituted with an alanine residue. These strains were analyzed for their ability to assemble a polar flagellum. Correctness of flagella assembly was analyzed by fluorescence microscopy after maleimide-staining of either the hook or filament structures, as described previously (Heimbrook et al., 1989). Depending on whether the filament or the hook was visualized, 67 or 56%, respectively, of the wild-type cells were flagellated, while the remaining cells were apparently non-flagellated (Figures 2A, 3B). This observation suggests that visualization of flagella through filament staining seems slightly more effective than with the hook staining, likely because of a more efficient staining of the significantly larger filament.

As expected from the essential nature of FlhB for flagellar biogenesis, deletion of *flhB* leads to completely non-flagellated cells independently of whether filaments or hooks were stained. (Figures 2A, 3A,B). Also the substitution of the cleavage site residue N269 by an alanine leads to the total absence of flagellar filaments, due to the absence of FlhB-C autocleavage into FlhB-CN and FlhB-CC, which ultimately is required for substrate specificity switching and therefore filament protein export. Moreover, our analysis shows that the ΔPRR and Y376A strains still exhibit one polar flagellum like the wild type, indicating that neither the entire PRR nor its C-terminal residue affect the spatial-numerical regulation of flagella.

However, unlike the strain carrying the Y376A substitution (filaments: 65 ± 4%; hooks: 59 ± 4%), the ΔPRR strain significantly differs from the wild type in the absolute number of flagellated cells. Our experiments show that the absence of the PRR results in a reduction of 31 ± 7 and 26 ± 10% in flagellation as determined by filament- and hook-staining, respectively (Figures 2A, 3B, respectively). Depending on whether the presence of flagella is visualized by filament or hook staining, a difference of approximately 5% is observed. This difference might originate in the deletion of the PRR causing an increase in export dysfunction as the growth of extracellular flagellar structures progresses, leading to the termination of flagellin export in a part of the cell population. These experiments on the one hand suggest that the PRR at the very C-terminus of FlhB contributes to the efficient assembly of flagella in *S. putrefaciens* and affects hook and filament assembly likely at the level of fT3SS-dependent secretion. We also analyzed the overall length of filaments of each FlhB mutant in the cells that still assembled a flagellar filament. We observed an overall decrease of filament length for the PRR mutant in comparison to both the wild type and the Y376A mutant, which exhibited a filamentation similar to the wild type (Figure 2B). Added to the previous observations, this furthermore supports the notion that the PRR of FlhB functionally serves at the level of an efficient export of hook and filament proteins.

### PRR Deletion and FlhB Cleavage Inhibition Do Not Interfere With FliK-Dependent Polyhooks

Previous studies showed that in *S. enterica* variations in N269 and P270 that abolish and reduce flagellin export, respectively, result in the formation of overly long hook structures, so-called polyhooks. We further assessed the role of the PRR, the cleavage site N269 and Y376 in the context of a *fliK* deletion strain. As shown for *S. enterica* (Williams et al., 1996), the absence of FliK also leads to the complete loss of filaments in all analyzed strains of *S. putrefaciens*. The formation of hooks in the individual mutant strains missing *fliK* did not differ quantitatively from the strains carrying a copy of *fliK* (Figure 3E). The microscopic analysis of these *fliK* deletion strains, however, showed an increased fluorescence signal (Figure 3C) in comparison to the wild type when the hook structure was visualized through staining. This strong increase in fluorescence was indicative for a formation of polyhooks, which previously had been observed in the context of a *fliK* deletion (Bange et al., 2010). To further assess the possible role of the PRR deletion in the formation of polyhooks, the overall size of flagellar hooks was determined by measuring the area of fluorescent foci, which indicate individual fluorescently tagged hooks. This analysis did not demonstrate any significant difference in hook size between the wild type and ΔPRR strain and the respective strains lacking also *fliK* (Figure 3D). Thus, the FliK phenotype occurs independently of the PRR. Correspondingly, neither the absence of the PRR, the autocleavage of FlhB or the variation of Y376 did not affect the general formation of the flagellar (poly)hooks. Thus, our study shows that the absence of the PRR results in a decrease of cells with hook structures independently of FliK.

### Flagellar C-Ring Assembly Occurs Independently of the FlhB PRR and Structurally Relevant Residues

**FIGURE 4:**
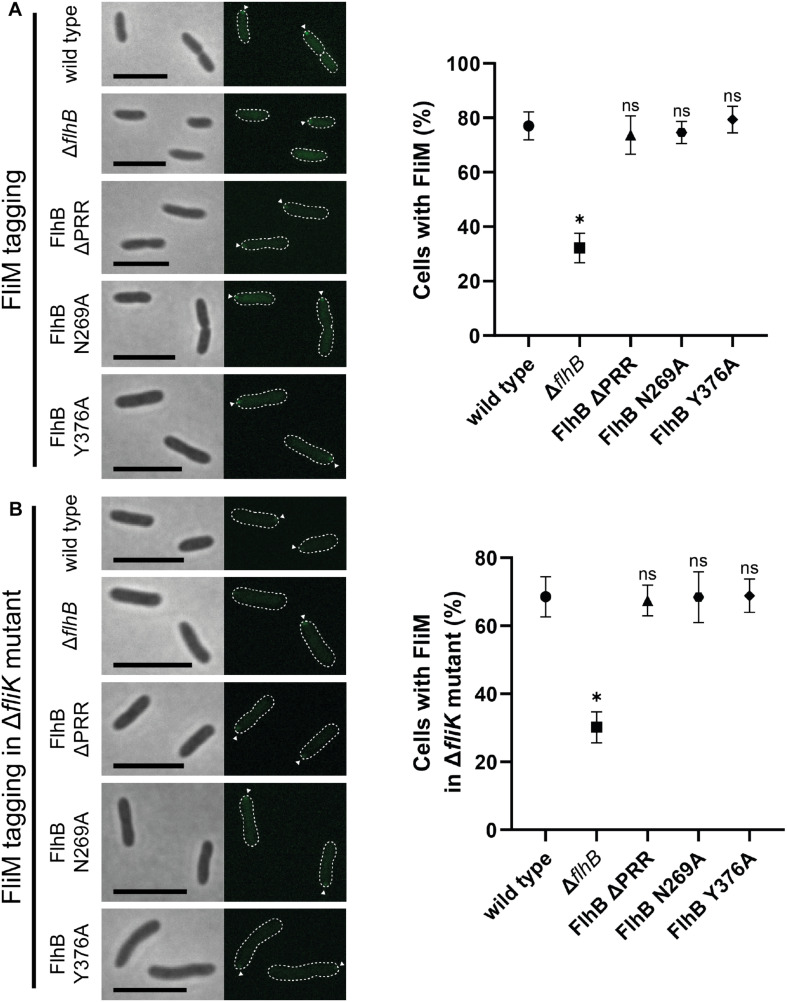
Microscopy and quantification analysis of the C-ring component FliM of *Sp*FlhB ΔPRR, N269A, and Y376A effects in comparison to wild type and a Δ*flhB* mutant in both a wild type and a Δ*fliK* background. **(A)** Microscopy images of FliM localization in cells and comparative quantification of FliM localization in FlhB mutant strains with appropriate controls in a wild type background. **(B)** Microscopy images of FliM localization in cells and comparative quantification of FliM localization in a Δ*fliK* background. Scale bar: 5 μm. Statistical analysis was conducted via unpaired *t*-test for each mutant in comparison to the wild type. * =̂ *P*-value ≤ 0.0001.

As an integral part of the fT3SS, FlhB is located in the center of the flagellar basal body adjacent to the flagellar C-ring. Thus, interactions between FlhB and C-ring components and involvement of FlhB in C-ring assembly appear possible. To verify such an involvement, we analyzed the localization behavior of the C-ring component FliM [FliM_1_ (Sputcn32_2569), henceforth simply FliM] in all the previously mentioned FlhB mutants. The quantification of FliM localization of each FlhB mutant relative to the wild type only showed a significant decrease in the Δ*flhB* mutant, indicating that neither the PRR, Y376, nor N269 play a role in C-ring assembly (Figure 4A). Further analysis of these strains carrying an additional deletion of *fliK* additionally showed FliM localization behavior, which, relative to the wild type, did not differ from the FlhB mutants carrying a copy of *fliK*, except for the overall amount of localizing FliM and foci strength (Figure 4B). This may be due to a similar effect caused by the deletion of *flhB* potentially destabilizing the cytoplasmic part of the flagellum in the absence of a crucial component. Hence we concluded that neither the entire PRR and its C-terminal residue Y376 nor the cleavage site residue N269 are required for C-ring assembly.

### FlhB Protein Abundance Is Affected by PRR Deletion and Modification of Cleavage Site (N269)

**FIGURE 5:**
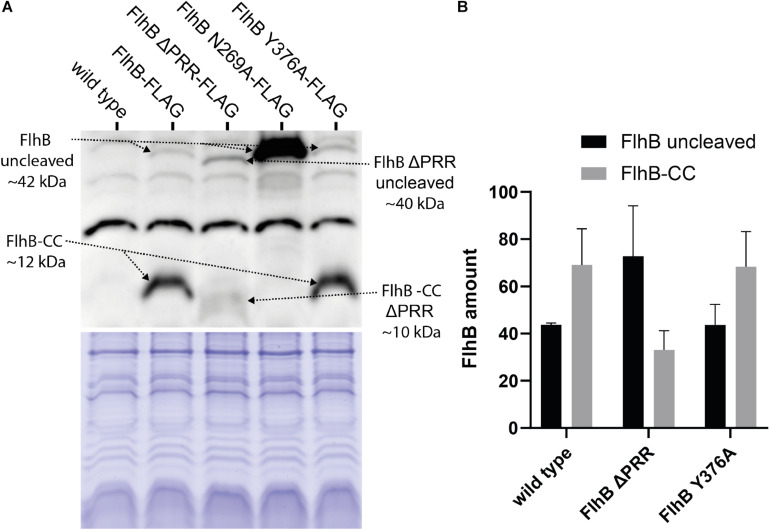
Western blot and protein amount quantification analysis of 3xFLAG tagged *Sp*FlhB wild type, FlhB ΔPRR, FlhB N269A, and FlhB Y376A. **(A)** Western blot and Coomassie-stained SDS-PAGE showing samples of each individual FlhB mutant strain with C-terminally 3xFLAG tagged FlhB detected by Monoclonal ANTI_FLAG M2-Peroxidase mouse antibodies. **(B)** Comparative mean gray value analysis of uncleaved FlhB and FlhB-CC. FlhB N269A was not included in the mean gray value analysis due to the excessive amount of uncleaved FlhB.

A plausible reason for our PRR-related phenotypes might simply be explainable by differences in the available amount of FlhB and its variants in the cellular context. Therefore, we constructed strains in which the corresponding *flhB* genes were modified on the chromosome to result in FlhB protein variants with a C-terminal 3xFLAG tag, which allows detecting FlhB (Figure 5A). These strains were then used to quantify the amounts of cleaved and non-cleaved FlhB and its variants by Western blotting using antibodies against the FLAG tags (Figure 5B). In cells producing wild type FlhB, the majority of the protein was found to be cleaved, while a FlhB carrying the N269A variations appeared at the size of the non-cleaved protein. The FlhB-Y376A variant was indistinguishable from that wild-type FlhB, indicating that the Y376 does not affect the autocleavage of the protein. In contrast, we observed a diminished FlhB autocleavage when the PRR was lacking. These data indicate that the PRR might play a role in the autocleavage of FlhB. Overall, our results show that the PRR of FlhB affects the number of cells displaying hooks and filaments, but not the assembly of the C-ring, and suggest that this motif might be involved in regulating the autocleavage of FlhB.

### Similar Proline-Rich C-Terminal Tails Occur Within FlhB Proteins of γ- and β-Proteobacteria

**FIGURE 6:**
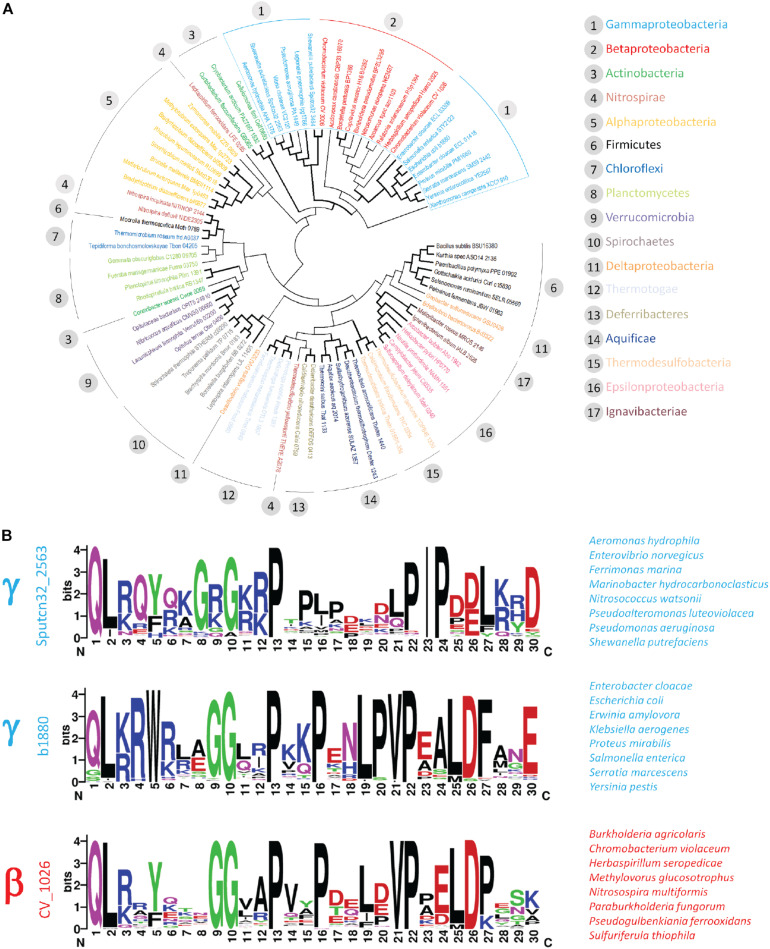
Occurrence of PRR within the γ- and β-proteobacteria. Phylogenetic analysis of representative FlhB sequences. Flagellar FlhB were distinguished from injectisome FlhB based on the classification provided by the KEGG database. Flagellar FlhB proteins are summarized in KEGG orthology K02401, while the functional and structural homologs from T3SS are summarized in KEGG orthology KO3229. **(A)** Bifurcations supported in more or equal 50% of bootstrap replicates are shown in bold. **(B)** Sequence logos of PRR identified in the two γ- and the β-proteobacterial cluster. The locus tag of the query sequence used to identify the PRR is depicted on the left and representative species are given on the right.

The intriguing clustering and structural arrangement of five proline residues within the C-terminal tail of the polar flagellar FlhB of *S. putrefaciens* prompted us to ask, whether PRRs frequently occur within members of the FlhB protein family. First, we performed a sequence comparison for a collection of representative FlhB proteins extracted from all major bacterial phyla (Supplementary Table S4). This analysis reflected the close relationship of γ- and β-proteobacteria (Williams et al., 2010) for FlhB but also revealed that FlhB proteins of γ- proteo-, β- proteo-, and Actinobacteria are usually characterized by a C-terminal extension of the FlhB domain (Pfam Bac_export_2) of about 30–60 amino acids (Supplementary Figure S2 and Figure 6). Closer inspection of these tail regions identified a clear accumulation of proline residues in FlhB of γ- but also β-proteobacteria. We also noted that proline residues frequently occur in the C-terminus of FlhB from Actinobacteria. However, since the arrangement and number of prolines differed considerably from the pattern observed in *S. putrefaciens*, they were not further considered here.

To explore the occurrence of the PRR within the β- and γ-proteobacteria in more detail, we performed BLAST searches using representative FlhB C-termini from the two major γ- and the related β-proteobacterial FlhB clusters identified in our phylogenetic analysis (Figure 6A). This analysis confirmed the presence of proline-rich motifs in several classes of the γ- and β-proteobacteria. The motif identified with the *S. putrefaciens* polar FlhB as query occurred specifically within the γ-proteobacteria (e.g., Alteromonadales, Vibrionales, Pseudomonales). Two very closely related proline-rich motifs were identified in the Enterobacterales order of the γ-proteobacteria and several groups of β-proteobacteria, including the Neisseriales, Burkholderiales, and Nitrosomonadales (Figure 6B). Although displaying some differences, common features can be identified for these three motifs: (i) the presence of a highly conserved N-terminal glycine residue, (ii) up to five proline residues separated by one to four variable amino acids, (iii) a valine or isoleucine residue preceding the highly conserved fourth and fifth proline in the β- and γ-proteobacteria, respectively, and (iv) negatively charged residues in close proximity of the last proline residue (Figure 6).

We would like to note that the PRR is not present in the FlhB homologs of injectisome-related type III secretion system. Taken together the PRR and its slight variations represent a unique feature of the FlhB proteins found in fT3SS of the β- and γ-proteobacteria.

## Discussion

In this study, we visualized a PRR localizing at the very C-terminus of the polar flagellar type III secretion protein FlhB of *S. putrefaciens*. Our phylogenetic analysis shows that the PRR is common within the flagellated beta and gamma-proteobacteria, and only rarely found in other bacterial phyla. Our structural analysis shows that the PRR exhibits a “wave”-shaped linear motif, which interacts with the FlhB-C core domain in a highly defined manner. Thus, it is plausible to think that the PRR either represents a distinct interaction site or masks a binding surface at the core domain for a yet to be identified partner of FlhB. This putative binding partner might be part of the fT3SS/basal body/C-ring and directly contribute to the efficiency of the secretion process. We wanted to note that the lateral FlhB, despite the secondary flagellar system being distinct and differently regulated compared to the polar one, also contains the PRR motif.

Deletion of the PRR in the polar *S. putrefaciens* FlhB leads to a reduction in the number of cells producing hook and filament structure in the polar flagellum; however, it does not affect formation of the flagellar C-ring as analyzed via the C-ring protein FliM. These observations suggest that the PRR could be involved in the assembly of extracellular flagellar structures following the basal body. Interestingly, the deletion of the FlhB PRR, even though it leads to a moderate reduction in autocleavage, appears not to mirror the filament formation phenotype of the FlhB N269A mutant, which is incapable of autocleavage. The significant reduction of hook forming cells observed in the ΔPRR mutant, in comparison to the wild type, which does not appear in the N269A mutant, additionally supports the notion of the PRR being involved in overall export efficiency of hook and filament proteins. Nonetheless, several scenarios of how the PRR of FlhB mechanistically contributes to these observations can be imagined.

Besides the newly identified PRR, we also show the conserved necessity of the previously described cleavage site N269 of FlhB (Meshcheryakov et al., 2013a) for correct flagellum assembly in *S. putrefaciens*. Our observation shows a complete absence of filaments in this mutant, indicating, as the formation of flagellar hooks is not inhibited, total abolishment of substrate specificity switching of the fT3SS export machinery. We therefore conclude that similarly to *Salmonella* sp. FlhB (Meshcheryakov et al., 2013a), the FlhB of *S. putrefaciens* requires the asparagine at position 269 for FlhB-C cleavage induced substrate specificity switching. The insignificant impact the substitution of the C-terminal PRR residue Y376 had on the overall formation of flagellar structures and the amount of FlhB protein points to a rather minor role in the establishment of general flagellation.

A key interaction partner of FlhB is FliK, a protein of two domains of different function that are associated with a long, natively unstructured linker region (Kodera et al., 2015). FliK is secreted during hook assembly (Minamino et al., 1999), and it has been shown that its N-terminal domain serves in determining the length of the hook structure. The C-terminal domain of FliK interacts with FlhB (Williams et al., 1996; Kinoshita et al., 2017). Recently, both the *in vivo* role of the PRR (residues 350/353–383 in *S. enterica*) and its *in vitro role* in binding flagellar secretion substrates were investigated in detail (Inoue et al., 2019). This work showed that there was a significant increase in motility with several *S. enterica* FlhB mutant strains (1-350, 1-355, and 1-360) in the absence, but not the presence of FliK. In addition, the FlhB 1-365, 1-360, and 1-355 mutant strains were shown to cause a tendency to variable greater hook length in a small part of the population, pointing toward a less effective hook length control mediated by the FlhB PRR/CCT segment. The same study showed that an overexpression of wild-type FlhB-C with C-terminal truncations ending at residue 375 or 380 resulted in more pronounced cleavage suggesting that residues 350–370 of the PRR could affect the conformation of the NPTH loop (and hence reduce the extent of cleavage).

Regarding the interaction of FlhB with flagellar secretion substrates, removal of the PRR did not impair the binding of the hook protein FlgD to FlhB in previous experiments (Evans et al., 2013). This study identified a hydrophobic patch of four amino acid residues that is involved in the recognition of flagellar secretion substrates. Our structure shows that none of these residues is located in the direct proximity of, or interacting with the PRR motif. Therefore, the PRR seems not to be directly involved in the binding of export substrates to FlhB.

An additional interaction partner of FlhB is FlhA, which (like FlhB) is a core component of the flagellar T3SS, and it can also be divided into TMD followed by a cytoplasmic domain (FlhA-C). FlhA-C provides the adaptor for the coordinated delivery of late flagella building blocks to the fT3SS (Bange et al., 2010). It interacts with the flagellar filament and filament-cap proteins flagellin and FliD when they are bound to their cognate chaperones FlS and FliT, respectively (Kinoshita et al., 2013; Altegoer et al., 2018; Xing et al., 2018). Additionally, FlhA can also interact with the MS-ring component FliF (Kihara et al., 2001), as well as the cytoplasmic domain of FlhB itself (Minamino et al., 2010). Interestingly, a previous study tested for interactions between a non-cleaver (N269A) FlhB-C and FlhA-C. They determined that the non-cleaver FlhB-C was unable to interact with FlhAc, but that it possibly requires functional cleavage to be able to do so (McMurry et al., 2004). Thus, it is conceivable that the PRR of FlhB might contribute to the interaction with FlhA-C. However, we could not observe a direct interaction of the isolated PRR with FlhA-C in our *in vitro* pulldown assays (data not shown). As for FlhA-C, we were unable to detect a direct interaction of the isolated PRR of FlhB with any part of FliK, suggesting that the PRR does not directly contribute to the FlhB/FliK interaction (data not shown). Furthermore, the absence of an exacerbated decrease in hook formation observed in the FlhB mutants lacking FliK, in comparison to the mutants carrying a copy of *fliK*, indicates that the loss of FliK does not add to the observed phenotypes in the context of export efficiency and therefore additionally suggests that besides the PRR mutant, the other analyzed FlhB residues are not required for FlhB/FliK interaction.

Summing up, FlhA and FliK are important interaction partners of FlhB that have an essential role in flagellar assembly. It has, however, not been shown that either of these proteins (or any additional interaction partners) are involved in a direct interaction with the far C-terminal region of Flhb-C, the PRR motif (in organisms where it is present). As mentioned earlier in the introduction, it is either possible that the PRR itself is a binding site, or that it otherwise blocks an interaction site on FlhB (for example, the sites identified in FlhB by Williams et al., 1996 are located relatively close N-terminally to the PRR motif). However, it is also possible that the PRR could represent a binding site for a yet to be identified regulatory factor, which impacts the assembly of the extracellular flagellar structures; e.g., in response to environmental signals. We now know that it is unlikely that the PRR is blocking the hydrophobic patch identified in Evans et al. (2013) as the interaction interface for hook/rod substrates. Therefore, future studies need to define the precise nature of a potentially unknown interaction partner and clarify the molecular consequences on hook and filament assembly in the beta- and gamma-proteobacteria.

## Materials and Methods

### Protein Expression and Purification for Crystallization

A pET24d vector was used for the protein construct that was overexpressed in *Escherichia coli* BL21 (DE3) competent cells. Cell cultures were grown in LB medium at 30°C overnight, and shaken at 180 r/min. 1% lactose monohydrate (weight/volume) was used for induction. Cells were harvested and lysed by microfluidizer (M110-L, Microfluidics), and centrifuged to pellet cell debris. The supernatant was then loaded onto a GE Healthcare HisTrapFF affinity column. The lysis and wash buffer contained 20 mM HEPES pH 8.0, 250 mM NaCl, 20 mM KCl, 20 mM MgCl2, 40 mM imidazole, while the imidazole concentration in the elution buffer was increased to 500 mM. After elution, the protein was purified by SEC using an S200 Sepharose column and GE Lifesciences AKTA Prime and Purifier systems. After purification, the proteins were concentrated using Amicon Ultra-15 spin concentrators (10 kDa cutoff point).

### Crystallization and Structure Determination

After the Ni-NTA affinity and SEC purification steps outlined in the previous paragraph, the FlhBc size exclusion peak was concentrated to a molar concentration of 0.5 mM. The mixture was then crystallized with the 96-needle Gryphon robot (Art Robbins), according to the manufacturer′s manual. For crystallization, JCSG core solutions I-IV and ProComplex (Qiagen) were used, together with two-well crystallization plates, suited for the sitting drop method (Swiss MRC). Crystals were later observed in three different conditions (JCSG Core III B2, 0.2 M lithium sulfate, 0.1 M Tris pH 8.5, 1.26 M ammonium sulfate; JCSG Core IV D5, 0.1 HEPES pH 7.5, 1.5 M lithium sulfate; Core ProComplex H6, 0.1 M MES pH 6.5, 1.6 M magnesium sulfate). The observed crystals, first detected 3 weeks after the crystallization attempt was carried out, were fished and frozen with cryoprotectant 4 weeks after crystallization in liquid nitrogen until measurement (only Core IV D5 and Core III B2 crystals were large enough to be harvested). The X-ray diffraction measurements were performed under cryogenic conditions at the DESY Synchrotron facility in Hamburg, Germany.

Data were processed with XDS and scaled with XSCALE (Kabsch, 2010). The structure was determined by molecular replacement with PHASER (McCoy et al., 2007), manually built in COOT (Emsley and Cowtan, 2004), and refined with PHENIX (Adams et al., 2010). The search model was the structure of *S. typhimurium* FlhBc (3B0Z) (Meshcheryakov et al., 2013b). Final validation of the structures was carried out with the validation server of the Protein Data Bank (PDB) at https://validate-rcsb-1.wwpdb.org (Gore et al., 2017). Figures were prepared with Pymol^1^. LigPlot + version 2.2 (Laskowski and Swindells, 2011) was used in order to analyze the interaction interface between the PRR and core domain of FlhB-C.

### Bacterial Strain Cultivation

Both *S. putrefaciens* and *E. coli* strains, which were used in this study, are compiled in Supplementary Table 1. LB medium was used as base medium for all cultivations, which for selection purposes was supplemented with either 50 mg/mL kanamycin or 10% (w/v) sucrose. The media for 2,6-diaminopimelinic acid (DAP)-auxotrophic *E. coli* WM3064 were supplemented with a final concentration of 300 μM DAP. Agarose pads for fluorescence microscopy were prepared by solidifying LM medium [10 mM HEPES, 100 mM NaCl, 0.02% (w/v) yeast, 0.01% (w/v) peptone, 10.5 mM lactate, pH 7.5] with 1% (w/v) agarose. *S. putrefaciens* and *E. coli* strains were grown at 30 and 37°C, respectively.

### Plasmid Construction

All plasmids and oligonucleotides used in this study are compiled in Supplementary Tables 2, 3. Construction of the Plasmids was done using the Gibson Assembly method (Gibson et al., 2009). Approximately 500–600 bp long fragments upstream and downstream of the gene of interest with up to 15 remaining nucleotides were utilized to generate markerless in-frame deletions. The FlhB mutants carrying either an amino acid substitution (N269A, Y376A), a truncation (Δ358–376 +) or a 3xFLAG tag, were generated by reintroducing the modified gene sequence into the Δ*flhB* strain. Fluorescent tagging of proteins was achieved via integrating the appropriate fluorophore gene with a linker, adjacent to the gene corresponding to the protein of interest, into the genome. To achieve all deletions and insertions the suicide vector pNPTS-138-R6K was linearized by *Eco*RV digestion.

### Strain Construction

*Shewanella putrefaciens* CN-32 was genetically modified to introduce none native alterations, such as partial or complete deletions and fluorescent fusions, into the genome. This was achieved through sequential double homologous recombination by utilizing the suicide plasmid pNPTS-138-R6K (Lassak et al., 2010). The conjugation strain *E. coli* WM3064 was used as the donor to introduce the individual plasmid into each respective *S. putreafaciens* CN-32 recipient strain.

### Fluorescent Filament and Hook Staining

Flagellar filament (flaB1 T166C flaA1 T174C; 38) and hook (flgE1 T183C) structures of exponentially growing cells were fluorescently labeled using Alex Fluor 488 maleimide and Alex Fluor 568 maleimide (Thermo Fisher Scientific) in accordance with previously publicized methods (Bubendorfer et al., 2012).

### Fluorescence Microscopy

Samples for microscopy imaging, of which 2 μL was spotted onto an LM-agarose pad, were harvested from exponential growing *S. putrefaciens* CN-32 cultures. Fluorescent microscopy images were acquired utilizing a DMI6000B inverse microscope (Leica) set up with a VisiScope Cell Explorer system (Visitron Systems GMBH) and an HCX PL APO 100x/1.4 PH3 phase contrast objective controlled by the VisiView software (Visitron Systems GmbH).

### SDS-PAGE and Western Blot

For the SDS-PAGE the method previously described by Laemmli (Lassak et al., 2010) was used. Samples were taken from exponentially growing cultures and set to OD 10 in 2xSDS sample buffer. 10 μL of each sample was loaded onto a 12.5% SDS gel. FlhB-3xFLAG proteins were detected with Monoclonal ANTI_FLAG M2-Peroxidase mouse antibodies (Sigma–Aldrich), the protein membrane was treated with Western Lightning^®^ Plus-ECL, Enhanced Chemiluminescence Substrate Kit (Perkin Elmer, United States) and developed for 1 min using a Fusion SL4 (peqlab, Darmstadt).

### Data Processing

Analysis (quantification and hook area measurement) and editing of images were done with ImageJ 1.52e (National Institutes of Health) and Adobe Illustrator CS6 16.0.3 (Adobe Systems Incorporated). Statistics and graph creation were conducted with Prism 8.3 (GraphPad Software). Filament length measurements and analysis were achieved with BacStalk 1.7stable (Universitaet Marburg) (Hartmann et al., 2020).

### Sequence Comparison of FlhB Proteins and PRR Identification

A set of 86 FlhB proteins from the KEGG^2^ orthology group K02401 (flagellar biosynthetic protein FlhB) representing all major bacterial phyla was used to construct a multiple sequence alignment using Muscle (Edgar, 2004). From this alignment, the relationship among FlhB proteins was inferred using the maximum likelihood method and Le and Gascuel amino acid substitution model (Le and Gascuel, 2008). The tree with the highest log likelihood (−53146.61) is shown in Figure 3A. Initial tree(s) for the heuristic search were obtained automatically by applying neighbor-join and BioNJ algorithms to a matrix of pairwise distances estimated using a JTT model, and then selecting the topology with superior log likelihood value. A discrete Gamma distribution was used to model evolutionary rate differences among sites [five categories (+G, parameter = 2.3939)]. The rate variation model allowed for some sites to be evolutionarily invariable ([+I], 0.75% sites). This analysis involved 86 amino acid sequences. There were a total of 467 positions in the final dataset. Evolutionary analyses were conducted in MEGA X (Kumar et al., 2018). 500 bootstrap replicates were calculated.

To investigate the occurrence of proline-rich FlhB C-termini within the γ- (taxid:1236)- and β-proteobacteria (taxid:28216), the NCBI microbial proteins data set was searched using the BLASTp function with the C-terminal 30 and 40 amino acids of FlhB from *S. putrefacience* (Sputcn32_2563) and *Chromobacterium violaceum* (CV_1026) as query, respectively. FlhB proteins identified with an E-value of <0.05 were downloaded, filtered for redundancy with skipredundant^3^ using a 90% identity cut-off and aligned with Muscle for the visualization of amino acid frequencies with WebLogo (Crooks and Brenner, 2004).

## Data Availability Statement

Structure factors and coordinates have been deposited at the Protein Data Base with the accession code: 6Z0W. Plasmids, primers, and strains are available on request.

## Author Contributions

VB, JH, DM, and LS performed the experiments. JP-F performed phylogenetic analysis. FA, VB, and GB determined the structure. KT and GB conceptualized the study and provided resources and funding. VB, JH, JP-F, KT, and GB wrote the manuscript. All authors read and commented on the manuscript.

## Conflict of Interest

The authors declare that the research was conducted in the absence of any commercial or financial relationships that could be construed as a potential conflict of interest.

## Footnotes

1 www.pymol.org

2 https://www.kegg.jp

3 https://www.bioinformatics.nl/cgi-bin/emboss/skipredundant
