# Supplementary material for: A Proline-Rich Element in the Type III Secretion Protein FlhB Contributes to Flagellar Biogenesis in the Beta- and Gamma-Proteobacteria
Source: Front Microbiol. 2020 Dec 15;11:564161. doi: 10.3389/fmicb.2020.564161 (PMC7771051; doi:10.3389/fmicb.2020.564161)
Supplement: Supplementary file 1 [file Data_Sheet_1.docx]

***Supplementary Information for:***

**A proline-rich element in the type III secretion protein FlhB contributes to flagellar biogenesis in the beta- and gamma-proteobacteria**

John C. Hook^2,3^, Vitan Blagotinsek^1,3^, Jan Pané-Farré^1^, Devid Mrusek^1^, Florian Altegoer^1^, Anita Dornes^1^,  MeikeSchwan^2^, Lukas Schier^1^, Kai M. Thormann^2^ and Gert Bange^1^

^1^Philipps-University Marburg, SYNMIKRO Research Center & Department of Chemistry, Hans-Meerwein-Strasse 6, C07, 35043 Marburg, Germany

^2^Justus-Liebig-Universität, Department of Microbiology and Molecular Biology, Heinrich-Buff-Ring 26, 35392 Giessen, Germany

^3^These authors contribute equally to the work.

Correspondence: [gert.bange@synmikro.uni-marburg.de](mailto:gert.bange@synmikro.uni-marburg.de), [Kai.Thormann@mikro.bio.uni-giessen.de](mailto:Kai.Thormann@mikro.bio.uni-giessen.de)

***This file contains:***

Supplementary Tables S1-S4

Supplementary Figures. S1-S2

**Supplementary Table S1.** Strains used in this study

| Strain | Genotype | Purpose | Reference |
| --- | --- | --- | --- |
| *Escherichia coli* strains |  |  |  |
| DH5α λpir | ϕ80d*lacZ* ΔM15 Δ(*lacZYA-argF*)U169 *recA1* *hsdR17 deoR* *thi-l supE44 gyrA96* *relA1*/λpir | cloning strain | (1) |
| WM3064 | *thrB1004 pro thi rpsL hsdS lacZ* ΔM15 RP4‐1360 Δ(*araBAD*) 567Δ*dapA* 1341::[*erm pir*(wt)] | conjugation strain for *Shewanella* | W. Metcalf, University of Illinois, Urbana‐Champaign |
| *Shewanella putrefaciens* CN-32 strains |  |  |  |
| S4003 | *flgE_1_* T183C | markerless in-frame substitution of Thr183 to Cys in the polar hook protein FlgE_1_ (Sputcn32_3465), fully functional and suitable for maleimide staining | (2) |
| S5910 | *flgE_1_* T183C Δ*flagL* | markerless in-frame substitution of Thr183 to Cys in the polar hook protein FlgE_1_ (Sputcn32_3465), fully functional and suitable for maleimide staining and deletion of the lateral gene cluster (S*putcn32_3444*-*Sputcn32_3485*) | this study |
| S6994 | *flgE_1_* T183C Δ*flagL* FliM_1_-1xGS-GFP | markerless in-frame substitution of Thr183 to Cys in the polar hook protein FlgE_1_ (Sputcn32_3465), fully functional and suitable for maleimide staining, deletion of the lateral gene cluster (S*putcn32_3444*-*Sputcn32_3485*) and C-terminal GFP tag of FliM_1_ (Sputcn32_2569) linked with Gly and Ser | this study |
| S7071 | *flgE_1_* T183C Δ*flagL* FliM_1_-1xGS-GFP Δ*fliK*_1_ | markerless in-frame substitution of Thr183 to Cys in the polar hook protein FlgE_1_ (Sputcn32_3465), fully functional and suitable for maleimide staining, deletion of the lateral gene cluster, C-terminal GFP tag of FliM_1_ (Sputcn32_2569) linked with Gly and Ser and deletion of *fliK* (*Sputcn32_2571*) | this study |
| S6306 | *flgE_1_* T183C Δ*flagL* Δ*flhB* | markerless in-frame substitution of Thr183 to Cys in the polar hook protein FlgE_1_ (Sputcn32_3465), fully functional and suitable for maleimide staining, deletion of the lateral gene cluster and deletion of the gene *flhB* (S*putcn32_2563*) | this study |
| S6995 | *flgE_1_* T183C Δ*flagL* Δ*flhB* FliM_1_-1xGS-GFP | markerless in-frame substitution of Thr183 to Cys in the polar hook protein FlgE_1_ (Sputcn32_3465), fully functional and suitable for maleimide staining, deletion of the lateral gene cluster (S*putcn32_3444*-*Sputcn32_3485*) and deletion of the gene *flhB* and C-terminal GFP tag of FliM_1_ (Sputcn32_2569) linked with Gly and Ser | this study |
| S7072 | *flgE_1_* T183C Δ*flagL* Δ*flhB* FliM_1_-1xGS-GFP Δ*fliK*_1_ | markerless in-frame substitution of Thr183 to Cys in the polar hook protein FlgE_1_ (Sputcn32_3465), fully functional and suitable for maleimide staining, deletion of the lateral gene cluster (S*putcn32_3444*-*Sputcn32_3485*), deletion of the gene *flhB*, C-terminal GFP tag of FliM_1_ (Sputcn32_2569) linked with Gly and Ser and deletion of *fliK* (*Sputcn32_2571*) | this study |
| S6328 | *flgE_1_* T183C Δ*flagL* Δ*flhB* FlhB Δ358-376^+^ | markerless in-frame substitution of Thr183 to Cys in the polar hook protein FlgE_1_ (Sputcn32_3465), fully functional and suitable for maleimide staining, deletion of the lateral gene cluster (S*putcn32_3444*-*Sputcn32_3485*) and deletion of the gene *flhB* (Sputcn32_2563) with reconstitution of *flhB* with a truncated version (FlhB Δ358-376) | this study |
| S6920 | *flgE_1_* T183C Δ*flagL* Δ*flhB* FlhB Δ358-376^+^ FliM_1_-1xGS-GFP | markerless in-frame substitution of Thr183 to Cys in the polar hook protein FlgE_1_ (Sputcn32_3465), fully functional and suitable for maleimide staining, deletion of the lateral gene cluster (S*putcn32_3444*-*Sputcn32_3485*), deletion of the gene *flhB* with reconstitution of *flhB* with a truncated version and C-terminal GFP tag of FliM_1_ (Sputcn32_2569) linked with Gly and Ser | this study |
| S7070 | *flgE_1_* T183C Δ*flagL* Δ*flhB* FlhB Δ358-376^+^ FliM_1_-1xGS-GFP Δ*fliK*_1_ | markerless in-frame substitution of Thr183 to Cys in the polar hook protein FlgE_1_ (Sputcn32_3465), fully functional and suitable for maleimide staining, deletion of the lateral gene cluster (S*putcn32_3444*-*Sputcn32_3485*), deletion of the gene *flhB* (Sputcn32_2563) with reconstitution of *flhB* with a truncated version (FlhB Δ358-376), C-terminal GFP tag of FliM_1_ (Sputcn32_2569) linked with Gly and Ser and deletion of *fliK* (*Sputcn32_2571*) | this study |
| S7094 | *flgE_1_* T183C Δ*flagL* Δ*flhB* FliM_1_-1xGS-GFP FlhB N269A | markerless in-frame substitution of Thr183 to Cys in the polar hook protein FlgE_1_ (Sputcn32_3465), fully functional and suitable for maleimide staining, deletion of the lateral gene cluster (S*putcn32_3444*-*Sputcn32_3485*) and deletion of the gene *flhB*, C-terminal GFP tag of FliM_1_ (Sputcn32_2569) linked with Gly and Ser and reconstitution of *flhB* with a mutated version (FlhB N269A) | this study |
| S7096 | *flgE_1_* T183C Δ*flagL* Δ*flhB* FliM_1_-1xGS-GFP Δ*fliK*_1_ FlhB N269A | markerless in-frame substitution of Thr183 to Cys in the polar hook protein FlgE_1_ (Sputcn32_3465), fully functional and suitable for maleimide staining, deletion of the lateral gene cluster (S*putcn32_3444*-*Sputcn32_3485*) and deletion of the gene *flhB*, C-terminal GFP tag of FliM_1_ (Sputcn32_2569) linked with Gly and Ser, deletion of *fliK* (*Sputcn32_2571*) and reconstitution of *flhB* with a mutated version (FlhB N269A) | this study |
| S7081 | *flgE_1_* T183C Δ*flagL* Δ*flhB* FliM_1_-1xGS-GFP FlhB Y376A | markerless in-frame substitution of Thr183 to Cys in the polar hook protein FlgE_1_ (Sputcn32_3465), fully functional and suitable for maleimide staining, deletion of the lateral gene cluster (S*putcn32_3444*-*Sputcn32_3485*) and deletion of the gene *flhB*, C-terminal GFP tag of FliM_1_ (Sputcn32_2569) linked with Gly and Ser and reconstitution of *flhB* with a mutated version (FlhB Y376A) | this study |
| S7083 | *flgE_1_* T183C Δ*flagL* Δ*flhB* FliM_1_-1xGS-GFP Δ*fliK*_1_ FlhB Y376A | markerless in-frame substitution of Thr183 to Cys in the polar hook protein FlgE_1_ (Sputcn32_3465), fully functional and suitable for maleimide staining, deletion of the lateral gene cluster (S*putcn32_3444*-*Sputcn32_3485*) and deletion of the gene *flhB*, C-terminal GFP tag of FliM_1_ (Sputcn32_2569) linked with Gly and Ser, deletion of *fliK* (*Sputcn32_2571*) and reconstitution of *flhB* with a mutated version (FlhB Y376A) | this study |
| S4401 | *flaB_1_* T166C *flaA*_1_ T174C Δ*flagL* | markerless in-frame substitution of Thr166 to Cys in the polar major flagellin protein FlaB_1_ (Sputcn32_2585) and Thr174 to Cys in the polar minor flagellin protein FlaA_1_ (Sputcn32_2586), fully functional and suitable for maleimide staining and deletion of the lateral gene cluster (S*putcn32_3444*-S*putcn32_3485*) | (3) |
| S7067 | *flaB_1_* T166C *flaA*_1_ T174C Δ*flagL* Δ*fliK*_1_ | markerless in-frame substitution of Thr166 to Cys in the polar major flagellin protein FlaB_1_ (Sputcn32_2585) and Thr174 to Cys in the polar minor flagellin protein FlaA_1_ (Sputcn32_2586), fully functional and suitable for maleimide staining, deletion of the lateral gene cluster (S*putcn32_3444*-S*putcn32_3485*) and deletion of *fliK* (*Sputcn32_2571*) | this study |
| S6305 | *flaB_1_* T166C *flaA*_1_ T174C Δ*flagL* Δ*flhB* | markerless in-frame substitution of Thr166 to Cys in the polar major flagellin protein FlaB_1_ (Sputcn32_2585) and Thr174 to Cys in the polar minor flagellin protein FlaA_1_ (Sputcn32_2586), fully functional and suitable for maleimide staining, deletion of the lateral gene cluster (S*putcn32_3444*-*Sputcn32_3485*) and deletion of the gene *flhB* (S*putcn32_2563*) | this study |
| S7068 | *flaB_1_* T166C *flaA*_1_ T174C Δ*flagL* Δ*flhB* Δ*fliK*_1_ | markerless in-frame substitution of Thr166 to Cys in the polar major flagellin protein FlaB_1_ (Sputcn32_2585) and Thr174 to Cys in the polar minor flagellin protein FlaA_1_ (Sputcn32_2586), fully functional and suitable for maleimide staining, deletion of the lateral gene cluster (S*putcn32_3444*-*Sputcn32_3485*), deletion of the gene *flhB* (S*putcn32_2563*) and deletion of *fliK* (*Sputcn32_2571*) | this study |
| S6327 | *flaB_1_* T166C *flaA*_1_ T174C Δ*flagL* Δ*flhB* FlhB Δ358-376^+^ | markerless in-frame substitution of Thr166 to Cys in the polar major flagellin protein FlaB_1_ (Sputcn32_2585) and Thr174 to Cys in the polar minor flagellin protein FlaA_1_ (Sputcn32_2586), fully functional and suitable for maleimide staining, deletion of the lateral gene cluster (S*putcn32_3444*-*Sputcn32_3485*) and deletion of the gene *flhB* (S*putcn32_2563*) with reconstitution of *flhB* with a truncated version (FlhB Δ358-376) | this study |
| S7069 | *flaB_1_* T166C *flaA*_1_ T174C Δ*flagL* Δ*flhB* FlhB Δ358-376^+^ Δ*fliK*_1_ | markerless in-frame substitution of Thr166 to Cys in the polar major flagellin protein FlaB_1_ (Sputcn32_2585) and Thr174 to Cys in the polar minor flagellin protein FlaA_1_ (Sputcn32_2586), fully functional and suitable for maleimide staining, deletion of the lateral gene cluster (S*putcn32_3444*-*Sputcn32_3485*), deletion of the gene *flhB* (S*putcn32_2563*) with reconstitution of *flhB* with a truncated version (FlhB Δ358-376) and deletion of *fliK* (*Sputcn32_2571*) | this study |
| S7093 | *flaB_1_* T166C *flaA*_1_ T174C Δ*flagL* Δ*flhB* FlhB N269A | markerless in-frame substitution of Thr166 to Cys in the polar major flagellin protein FlaB_1_ (Sputcn32_2585) and Thr174 to Cys in the polar minor flagellin protein FlaA_1_ (Sputcn32_2586), fully functional and suitable for maleimide staining, deletion of the lateral gene cluster (S*putcn32_3444*-*Sputcn32_3485*) and deletion of the gene *flhB* (S*putcn32_2563*) with reconstitution of *flhB* with a mutated version (FlhB N269A) | this study |
| S7095 | *flaB_1_* T166C *flaA*_1_ T174C Δ*flagL* Δ*flhB* Δ*fliK*_1_ FlhB N269A | markerless in-frame substitution of Thr166 to Cys in the polar major flagellin protein FlaB_1_ (Sputcn32_2585) and Thr174 to Cys in the polar minor flagellin protein FlaA_1_ (Sputcn32_2586), fully functional and suitable for maleimide staining, deletion of the lateral gene cluster (S*putcn32_3444*-*Sputcn32_3485*), deletion of the gene *flhB* (S*putcn32_2563*) with reconstitution of *flhB* with a mutated version (FlhB N269A) and deletion of *fliK* (*Sputcn32_2571*) | this study |
| S7080 | *flaB_1_* T166C *flaA*_1_ T174C Δ*flagL* Δ*flhB* FlhB Y376A | markerless in-frame substitution of Thr166 to Cys in the polar major flagellin protein FlaB_1_ (Sputcn32_2585) and Thr174 to Cys in the polar minor flagellin protein FlaA_1_ (Sputcn32_2586), fully functional and suitable for maleimide staining, deletion of the lateral gene cluster (S*putcn32_3444*-*Sputcn32_3485*) and deletion of the gene *flhB* (S*putcn32_2563*) with reconstitution of *flhB* with a mutated version (FlhB Y376A) | this study |
| S7082 | *flaB_1_* T166C *flaA*_1_ T174C Δ*flagL* Δ*flhB* Δ*fliK*_1_ FlhB Y376A | markerless in-frame substitution of Thr166 to Cys in the polar major flagellin protein FlaB_1_ (Sputcn32_2585) and Thr174 to Cys in the polar minor flagellin protein FlaA_1_ (Sputcn32_2586), fully functional and suitable for maleimide staining, deletion of the lateral gene cluster (S*putcn32_3444*-*Sputcn32_3485*), deletion of the gene *flhB* (S*putcn32_2563*) with reconstitution of *flhB* with a mutated version (FlhB Y376A) and deletion of *fliK* (*Sputcn32_2571*) | this study |

**Supplementary Table S2.** Plasmids used in this study

| Plasmid | Genotype | Reference |
| --- | --- | --- |
| pNPTS138-R6KT | *mob*RP4^+^ *ori*-R6K *sacB*, suicide plasmid for in frame deletions in *Shewanella putrefaciens*, Km^r^ | (4) |
| In-frame deletions |  |  |
| pNPTS138-R6KT Δ*flhB* | *flhB* (S*putcn32_2563*) in frame deletion suicide vector | this study |
| pNPTS138-R6KT Δ*flagL* | lateral gene cluster (*Sputcn32_3444-3485*) in frame deletion suicide vector | this study |
| pNPTS138-R6KT Δ*fliK*_1_ | *fliK* (*Sputcn32_2571*) in frame deletion suicide vector | this study |
| In-frame insertions |  |  |
| pNPTS138-R6KT FlhB Δ358-376^+^ | FlhB mutant (Δ358-367) in frame insertion suicide vector | this study |
| pNPTS138-R6KT FliM_1_-1xGS-GFP | C-terminal GFP tag of FliM_1_ (Sputcn32_2569) linked with Gly and Ser in frame insertion suicide vector | this study |
| pNPTS138-R6KT FlhB-3xFLAG | C-terminal 3xFLAG tag of FlhB (Sputcn32_2563) in frame insertion suicide vector | this study |
| pNPTS138-R6KT FlhB Δ358-376-3xFLAG | C-terminal 3xFLAG tag of FlhB (Sputcn32_2563) Δ358-376 mutant in frame insertion suicide vector | this study |
| pNPTS138-R6KT FlhB N269A-3xFLAG | C-terminal 3xFLAG tag of FlhB (Sputcn32_2563) N269A mutant in frame insertion suicide vector | this study |
| pNPTS138-R6KT FlhB Y376A-3xFLAG | C-terminal 3xFLAG tag of FlhB (Sputcn32_2563) Y376A mutant in frame insertion suicide vector | this study |

**Supplementary Table S3.** Oligonucleotides used in this study

| Plasmid | Identifier | Primer | Sequence |
| --- | --- | --- | --- |
| pNPTS138-R6KT Δ*flagL* | B25 | EcoRI-latFLAclus-fwd | A GAA TTC TGT GGT ATT GCC TCA CTC GCC |
|  | B26 | OL-latFLAclus-rev | CTA TGT CTC TGT CTG ATC AAA CAT TCT ATT CTG C |
|  | B27 | OL-latFLAclus-fwd | GAT CAG ACA GAG ACA TAG ATC CCT CTC GCG |
|  | B28 | PspOMI-latFLAclus-rev | T GTC GGG CCC ATT GCA CCA CAA TCA CAT CGG C |
|  | B29 | Check-latFLAclus-fwd | AAC GGT GTT CAA ATC GCC CAG |
|  | B30 | Check-latFLAclus-rev | AAT CCA ATG TTT GAG CAC GGC G |
| pNPTS138-R6KT Δ*flhB* | JH77 | EcoRV-flhB KO-fwd | GCC AAG CTT CTC TGC AGG AT GCG ATG GGG TTT GTT TCC CAG A |
|  | JH78 | OL-flhB KO-rev | TTT AAT GGG CTG CTC TCT TCA GCC ATA CTG AG |
|  | JH79 | OL-flhB KO-fwd | AGA GAG CAG CCC ATT AAA TCA GCC TAT CCC TGA TGA TTT AAA ATA TTA ATG |
|  | JH80 | EcoRV-flhB KO-rev | GCG AAT TCG TGG ATC CAG AT TCC ATC GCA CCA TAG AAA TCT GC |
|  | JH27 | fliQ1-fliR1 fw | GCA GTG TTT CAA GCT GCC ACT T |
|  | JH81 | Check-flhB KO-rev | GCA AAT TCC AGG GCT ATT GCT G |
| pNPTS138-R6KT Δ*fliK*_1_ | JH84 | EcoRV-fliK KO-fwd | GCC AAG CTT CTC TGC AGG AT GAT GGC GAA TGC AGA TCC CTT A |
|  | JH85 | OL-fliK KO-rev | ACC AGA ATG CAT TTG TTG CAT ATC AGC TCC CAA ACC |
|  | JH86 | OL-fliK KO-fwd | CAA CAA ATG CAT TCT GGT ATA GAT TAT TAC GCT TAA GC |
|  | JH87 | EcoRV-fliK KO-rev | GCG AAT TCG TGG ATC CAG AT CCT TAA CCT CTT TAA GGT ACT GAA ATT ACG |
|  | JH11 | fliI1-fliJ1 fw | GCG ATG AAT GCC TTC TTA AGG C |
|  | JH88 | Check-fliK KO-rev | TCA AGA TCA TCA TCG TCA TCG ACG |
| pNPTS138-R6KT FliM_1_-1xGS-GFP | JH98 | EcoRV FliM1 N-term fwd | GCG AAT TCG TGG ATC CAG AT GCT CAT TGA AGA TGC TCT CCT G |
|  | JH104 | OL FliM1 GFP rev | GAA AAG TTC TTC TCC TTT GCT GCT GCC TAA TTC AGA TAT ATC TCT AGC TTT GCC TTT GC |
|  | JH425 | OL-GFP-fwd | AGC AAA GGA GAA GAA CTT TTC |
|  | JH426 | OL-GFP-rev | G GAT CCT TTG TAG AGC TCA TCC |
|  | JH105 | OL FliM1 GFP fwd | GGA TGA GCT CTA CAA AGG ATC C TAA GGT GAA GCA AGA TGA GCA CAG AAG ATA |
|  | JH101 | EcoRV FliM1 N-term rev | GCC AAG CTT CTC TGC AGG AT AAT AAA ACT GCG GCC CAC TTC C |
|  | JH102 | Check-GFP FliM1-fwd | GCA GTT CAG ATG AGT CAT CCT C |
|  | JH103 | Check-GFP FliM1 KO-rev | GAC ATT TTG GCA GTT GAT GCG AC |
|  | JH98 | EcoRV FliM1 N-term fwd | GCG AAT TCG TGG ATC CAG AT GCT CAT TGA AGA TGC TCT CCT G |
| pNPTS138-R6KT FlhB Δ358-376^+^ | MS352 | EcoRV-FlhB-fwd | GCG AAT TCG TGG ATC CAG ATT TGC GAT GGG GTT TGT TTC CCA |
|  | MS353 | OL-FlhB KO C20AS rev | AAG CCA TTA CCG TCC CTT TTG GTA TTG ACG C |
|  | MS354 | OL-FlhB KO C20AS-fwd | AAG GGA CGG TAA TGG CTT GCT CGT TCA CTC TTC |
|  | MS355 | EcoRV-FlhB-rev | GCC AAG CTT CTC TGC AGG ATA ATC GCC ATT TGC TTA CCA GGC |
|  | JH27 | fliQ1-fliR1 fw | GCA GTG TTT CAA GCT GCC ACT T |
|  | JH81 | Check-flhB KO-rev | GCA AAT TCC AGG GCT ATT GCT G |
| pNPTS138-R6KT FlhB N269A/ FlhB N269A-3xFLAG | JH642 | EcoRV-FlhB N269A-fwd | GCG AAT TCG TGG ATC CAG AT GCG ATG GGG TTT GTT TCC CAG A |
|  | JH643 | OL FlhB N269A rev | ATA ATG CTC AGG GGC GAC GAC AAT C |
|  | JH644 | OL FlhB N269A fwd | GTG ATT GTC GTC GCC CCT GAG CAT |
|  | JH645 | EcoRV-FlhB N269A-rev | GCC AAG CTT CTC TGC AGG AT TCC ATC GCA CCA TAG AAA TCT GC |
|  | JH646 | Check FlhB N269A fwd | GCC ATG GCG ATT ACC TTT GCA A |
|  | JH640 | Check FlhB C-term rev | ACT CAC CTC TGC AAT ACG ACC A |
| pNPTS138-R6KT FlhB Y376A | JH642 | EcoRV-flhB KO-fwd | GCG AAT TCG TGG ATC CAG AT GCG ATG GGG TTT GTT TCC CAG A |
|  | JH647 | OL FlhB Y376A rev | ACG AGC AAG CCA TTA CGC TTT TAA ATC ATC |
|  | JH648 | OL FlhB Y376A fwd | CCT GAT GAT TTA AAA GCG TAA TGG CTT GCT |
|  | JH645 | EcoRV-flhB KO-rev | GCC AAG CTT CTC TGC AGG AT TCC ATC GCA CCA TAG AAA TCT GC |
|  | JH646 | Check FlhB N269A fwd | GCC ATG GCG ATT ACC TTT GCA A |
|  | JH640 | Check FlhB C-term rev | ACT CAC CTC TGC AAT ACG ACC A |
| pNPTS138-R6KT FlhB-3xFLAG | JH655 | EcoRV FlhB C-term fwd | GCG AAT TCG TGG ATC CAG AT GAT TGT GGT CAT TGA TGT GCC A |
|  | JH656 | OL FlhB FLAG rev | AAT ATC ATG ATC TTT ATA ATC GCC ATC ATG ATC TTT ATA ATC ATA TTT TAA ATC ATC AGG GAT AGG C |
|  | JH657 | OL FlhB FLAG fwd | ATT ATA AAG ATC ATG ATA TTG ATT ATA AAG ATG ATG ATG ATA AA TAA TGG CTT GCT CGT TCA CTC TT |
|  | JH638 | EcoRV FlhB C-term rev | GCC AAG CTT CTC TGC AGG AT GGA TGA TAA ATA CCA CTA AAC CCA C |
|  | JH658 | Check FlhB C-term fwd | CAC CAT GCG TTA GAT CTG TTG A |
|  | JH659 | Check FlhB C-term rev | ACT CAC CTC TGC AAT ACG ACC A |
| pNPTS138-R6KT FlhB Δ358-376^+^-3xFLAG | JH649 | EcoRV-FlhB dPRR-FLAG-fwd | GCG AAT TCG TGG ATC CAG AT GCG ATG GGG TTT GTT TCC CAG A |
|  | JH641 | OL FlhB Δ358-376-FLAG rev | AAT ATC ATG ATC TTT ATA ATC GCC ATC ATG ATC TTT ATA ATC CCG TCC CTT TTG GTA TTG ACG CAA |
|  | JH637 | OL FlhB FLAG fwd | ATT ATA AAG ATC ATG ATA TTG ATT ATA AAG ATG ATG ATG ATA AA TAA TGG CTT GCT CGT TCA CTC TT |
|  | JH638 | EcoRV FlhB C-term rev | GCC AAG CTT CTC TGC AGG AT GGA TGA TAA ATA CCA CTA AAC CCA C |
|  | JH646 | Check FlhB N269A fwd | GCC ATG GCG ATT ACC TTT GCA A |
|  | JH640 | Check FlhB C-term rev | ACT CAC CTC TGC AAT ACG ACC A |
| pNPTS138-R6KT FlhB Y376A-3xFLAG | JH649 | EcoRV-FlhB dPRR-FLAG-fwd | GCG AAT TCG TGG ATC CAG AT GCG ATG GGG TTT GTT TCC CAG A |
|  | JH635 | EcoRV FlhB C-term fwd | GCG AAT TCG TGG ATC CAG AT GAT TGT GGT CAT TGA TGT GCC A |
|  | JH636 | OL FlhB Y376A FLAG rev | AAT ATC ATG ATC TTT ATA ATC GCC ATC ATG ATC TTT ATA ATC CGC TTT TAA ATC ATC AGG GAT AGG C |
|  | JH637 | OL FlhB FLAG fwd | ATT ATA AAG ATC ATG ATA TTG ATT ATA AAG ATG ATG ATG ATA AA TAA TGG CTT GCT CGT TCA CTC TT |
|  | JH638 | EcoRV FlhB C-term rev | GCC AAG CTT CTC TGC AGG AT GGA TGA TAA ATA CCA CTA AAC CCA C |
|  | JH646 | Check FlhB N269A fwd | GCC ATG GCG ATT ACC TTT GCA A |
|  | JH640 | Check FlhB C-term rev | ACT CAC CTC TGC AAT ACG ACC A |
|  | JH639 | Check FlhB C-term fwd | CAC CAT GCG TTA GAT CTG TTG A |

**Supplementary Table S4.** FlhB sequences used in figure 3a in this study

| species name | FlhB locus tag (KEGG) | phylum | order | family |
| --- | --- | --- | --- | --- |
| Aquifex aeolicus VF5 | aq_2014 | Aquificae | Aquificales | Aquificaceae |
| Thermocrini salbus DSM14484 | Thal_1133 | Aquificae | Aquificales | Aquificaceae |
| Sulfurihydrogenibium azorense Az-Fu1 | SULAZ_1357 | Aquificae | Aquificales | Hydrogenothermaceae |
| Thermovibrio ammonificans HB-1 | Theam_1440 | Aquificae | Desulfurobacteriales | Desulfurobacteriaceae |
| Desulfurobacterium thermolithotrophum DSM11699 | Dester_1243 | Aquificae | Desulfurobacteriales | Desulfurobacteriaceae |
| Thermodesulfobacterium geofontis OPF15 | TOPB45_1330 | Thermodesulfobacteria | Thermodesulfobacteriales | Thermodesulfobacteriaceae |
| Thermodesulfatator indicus DSM15286 | Thein_0186 | Thermodesulfobacteria | Thermodesulfobacteriales | Thermodesulfobacteriaceae |
| Caldimicrobium thiodismutans TF1 | THC_0354 | Thermodesulfobacteria | Thermodesulfobacteriales | Thermodesulfobacteriaceae |
| Thermosipho melanesiensis BI429 | Tmel_0849 | Thermotogae | Thermotogales | Fervidobacteriaceae |
| Fervidobacterium nodosum Rt17-B1 | Fnod_0960 | Thermotogae | Thermotogales | Fervidobacteriaceae |
| Petrotoga mobilis SJ95 | Pmob_1397 | Thermotogae | Petrotogales | Petrotogaceae |
| Defluviitoga tunisiensis L3 | DTL3_1607 | Thermotogae | Petrotogales | Petrotogaceae |
| Thermomicrobium roseum DSM5159 | trd_A0037 | Chloroflexi | Thermomicrobiales | Thermomicrobiaceae |
| Tepidiforma bonchosmolovskayae 3753O | Tbon_04205 | Chloroflexi | Tepidiformia | Tepidiformales |
| Borreliella burgdorferi B31 | BB_0272 | Spirochaetes | Spirochaetales | Borreliaceae |
| Treponema pallidum subsp. Pallidum Nichols | TP_0715 | Spirochaetes | Spirochaetales | Spirochaetaceae |
| Spirochaeta thermophila DSM6192 | STHERM_c09290 | Spirochaetes | Spirochaetales | Spirochaetaceae |
| Leptospira interrogans serovar Linhai 56609 | LIL_11495 | Spirochaetes | Leptospirales | Leptospiraceae |
| Brachyspira murdochii DSM12563 | Bmur_0183 | Spirochaetes | Brachyspirales | Brachyspiraceae |
| Ignavibacterium album JCM 16511 | IALB_2526 | Ignavibacteriae | Ignavibacteria | Ignavibacteriales |
| Melioribacter roseus P3M-2 | MROS_2146 | Ignavibacteriae | Ignavibacteria | Ignavibacteriales |
| Calditerrivibrio nitroreducens DSM 19672 | Calni_0759 | Deferribacteres | Deferribacterales | Deferribacteraceae |
| Deferribacter desulfuricans SSM1 | DEFDS_0413 | Deferribacteres | Deferribacterales | Deferribacteraceae |
| Gemmata obscuriglobus DSM 5831 | C1280_09705 | Planctomycetes | Planctomycetia | Gemmatales |
| Fuerstia marisgermanicae NH11 | Fuma_03750 | Planctomycetes | Planctomycetia | Planctomycetales |
| Planctopirus limnophila DSM 3776 | Plim_1391 | Planctomycetes | Planctomycetia | Planctomycetales |
| Rhodopirellula baltica SH 1 | RB1347 | Planctomycetes | Planctomycetia | Pirellulales |
| Nibricoccus aquaticus HZ-65 | CMV30_06660 | Verrucomicrobia | Opitutae | Opitutales |
| Opitutaceae bacterium TAV5 | OPIT5_24910 | Verrucomicrobia | Opitutae | Opitutales |
| Opitutus terrae PB90-1 | Oter_0406 | Verrucomicrobia | Opitutae | Opitutales |
| Lacunisphaera limnophila | Verru16b_02200 | Verrucomicrobia | Opitutae | Opitutales |
| Cellulomonas fimi ATCC 484 | Celf_0693 | Actinobacteria | Micrococcales | Cellulomonadaceae |
| Conexibacter woesei DSM 14684 | Cwoe_0069 | Actinobacteria | Thermoleophilia | Solirubrobacterales |
| Curtobacterium flaccumfaciens pv. flaccumfaciens P990 | GBG65_16060 | Actinobacteria | Micrococcales | Microbacteriaceae |
| Cryobacterium arcticum PAMC 27867 | PA27867_1532 | Actinobacteria | Micrococcales | Microbacteriaceae |
| Kurthia sp. 11kri321 | ASO14_2135 | Firmicutes | Bacilli | Bacillales |
| Bacillus subtilis subsp. subtilis 168 | BSU16380 | Firmicutes | Bacilli | Bacillales |
| Gottschalkia acidurici 9a | Curi_c15830 | Firmicutes | Tissierellia | Tissierellales |
| Pelosnus fermentans JBW45 | JBW_01983 | Firmicutes | Negativicutes | Selenomonadales |
| Moorella thermoacetica ATCC 39073 | Moth_0789 | Firmicutes | Clostridia | Thermoanaerobacterales |
| Paenibacillus polymyxa E681 | PPE_01902 | Firmicutes | Bacilli | Bacillales |
| Selenomonas ruminantium subsp. lactilytica TAM6421 | SELR_05560 | Firmicutes | Negativicutes | Selenomonadales |
| Leptospirillum ferrooxidans C2-3 | LFE_0285 | Nitrospirae | Nitrospirales | Nitrospiraceae |
| Nitrospira defluvii | NIDE2305 | Nitrospirae | Nitrospirales | Nitrospiraceae |
| Candidatus Nitrospira inopinata | NITINOP_2144 | Nitrospirae | Nitrospirales | Nitrospiraceae |
| Thermodesulfovibrio yellowstonii DSM 11347 | THEYE_A2076 | Nitrospirae | Nitrospirales | Nitrospiraceae |
| Aeromonas hydrophila subsp. hydrophila ATCC 7966 | AHA_1378 | Proteobacteria | Gammaproteobacteria | Aeromonadales |
| Shewanella putrefaciens CN-32 | Sputcn32_2563 | Proteobacteria | Gammaproteobacteria | Alteromonadales |
| Shewanella putrefaciens CN-33 | Sputcn32_3484 | Proteobacteria | Gammaproteobacteria | Alteromonadales |
| Escherichia coli K-12 MG1655 | b1880 | Proteobacteria | Gammaproteobacteria | Enterobacterales |
| Enterobacter cloacae subsp. cloacae ATCC 13047 | ECL_01418 | Proteobacteria | Gammaproteobacteria | Enterobacterales |
| Enterobacter cloacae subsp. cloacae ATCC 13047 | ECL_03309 | Proteobacteria | Gammaproteobacteria | Enterobacterales |
| Proteus mirabilis HI4320 | PMI1660 | Proteobacteria | Gammaproteobacteria | Enterobacterales |
| Serratia marcescens SM39 | SM39_2442 | Proteobacteria | Gammaproteobacteria | Enterobacterales |
| Salmonella enterica subsp. enterica serovar Typhi CT18 | STY2123 | Proteobacteria | Gammaproteobacteria | Enterobacterales |
| Yersinia enterocolitica subsp. enterocolitica 8081 | YE2567 | Proteobacteria | Gammaproteobacteria | Enterobacterales |
| Legionella pneumophila subsp. pneumophila Philadelphia 1 | lpg1786 | Proteobacteria | Gammaproteobacteria | Legionellales |
| Pseudomonas aeruginosa PAO1 | PA1449 | Proteobacteria | Gammaproteobacteria | Pseudomonadales |
| Vibrio cholerae O1 El Tor N16961 | VC2120 | Proteobacteria | Gammaproteobacteria | Vibrionales |
| Xanthomonas campestris pv. campestris ATCC 33913 | XCC1910 | Proteobacteria | Gammaproteobacteria | Xanthomonadales |
| Bordetella pertussis Tohama I | BP1366 | Proteobacteria | Betaproteobacteria | Burkholderiales |
| Burkholderia pseudomallei K96243 | BPSL3295 | Proteobacteria | Betaproteobacteria | Burkholderiales |
| Cupriavidus necator H16 | H16_B0252 | Proteobacteria | Betaproteobacteria | Burkholderiales |
| Ralstonia solanacearum GMI1000 | RSp1394 | Proteobacteria | Betaproteobacteria | Burkholderiales |
| Acidovorax carolinensis NA2 | CBP33_16070 | Proteobacteria | Betaproteobacteria | Burkholderiales |
| Herbaspirillum seropedicae SmR1 | Hsero_2025 | Proteobacteria | Betaproteobacteria | Burkholderiales |
| Chromobacterium violaceum ATCC 12472 | CV_1026 | Proteobacteria | Betaproteobacteria | Neisseriales |
| Chromobacterium violaceum ATCC 12472 | CV_3006 | Proteobacteria | Betaproteobacteria | Neisseriales |
| Nitrosomonas europaea ATCC 19718 | NE2487 | Proteobacteria | Betaproteobacteria | Nitrosomonadales |
| Azoarcus sp. BH72 | azo1103 | Proteobacteria | Betaproteobacteria | Rhodocyclales |
| Bdellovibrio bacteriovorus HD100 | Bd3322 | Proteobacteria | Oligoflexia | Bdellovibrionales |
| Arcobacter butzleri RM4018 | Abu_1942 | Proteobacteria | Epsilonproteobacteria | Campylobacterales |
| Campylobacter jejuni subsp. jejuni NCTC 11168 | Cj0335 | Proteobacteria | Epsilonproteobacteria | Campylobacterales |
| Helicobacter pylori 26695 | HP0770 | Proteobacteria | Epsilonproteobacteria | Campylobacterales |
| Sulfurospirillum deleyianum DSM 6946 | Sdel_0240 | Proteobacteria | Epsilonproteobacteria | Campylobacterales |
| Nautilia profundicola AmH | NAMH_1551 | Proteobacteria | Epsilonproteobacteria | Nautiliales |
| Desulfovibrio vulgaris Hildenborough | DVU3233 | Proteobacteria | Deltaproteobacteria | Desulfovibrionales |
| Geobacter sulfurreducens PCA | GSU0426 | Proteobacteria | Deltaproteobacteria | Desulfuromonadales |
| Bradyrhizobium diazoefficiens USDA 110 | bll5809 | Proteobacteria | Alphaproteobacteria | Rhizobiales |
| Bradyrhizobium diazoefficiens USDA 111 | bll6877 | Proteobacteria | Alphaproteobacteria | Rhizobiales |
| Brucella melitensis bv. 1 16M | BMEII1114 | Proteobacteria | Alphaproteobacteria | Rhizobiales |
| Methylorubrum extorquens AM1 | Mex_1p0463 | Proteobacteria | Alphaproteobacteria | Rhizobiales |
| Methylorubrum extorquens AM1 | Mex_1p2723 | Proteobacteria | Alphaproteobacteria | Rhizobiales |
| Rhizobium leguminosarum bv. viciae 3841 | RL0699 | Proteobacteria | Alphaproteobacteria | Rhizobiales |
| Sinorhizobium meliloti 1021 | SMc03018 | Proteobacteria | Alphaproteobacteria | Rhizobiales |
| Zymomonas mobilis subsp. mobilis ATCC 29191 | ZZ6_0629 | Proteobacteria | Alphaproteobacteria | Sphingomonadales |

**
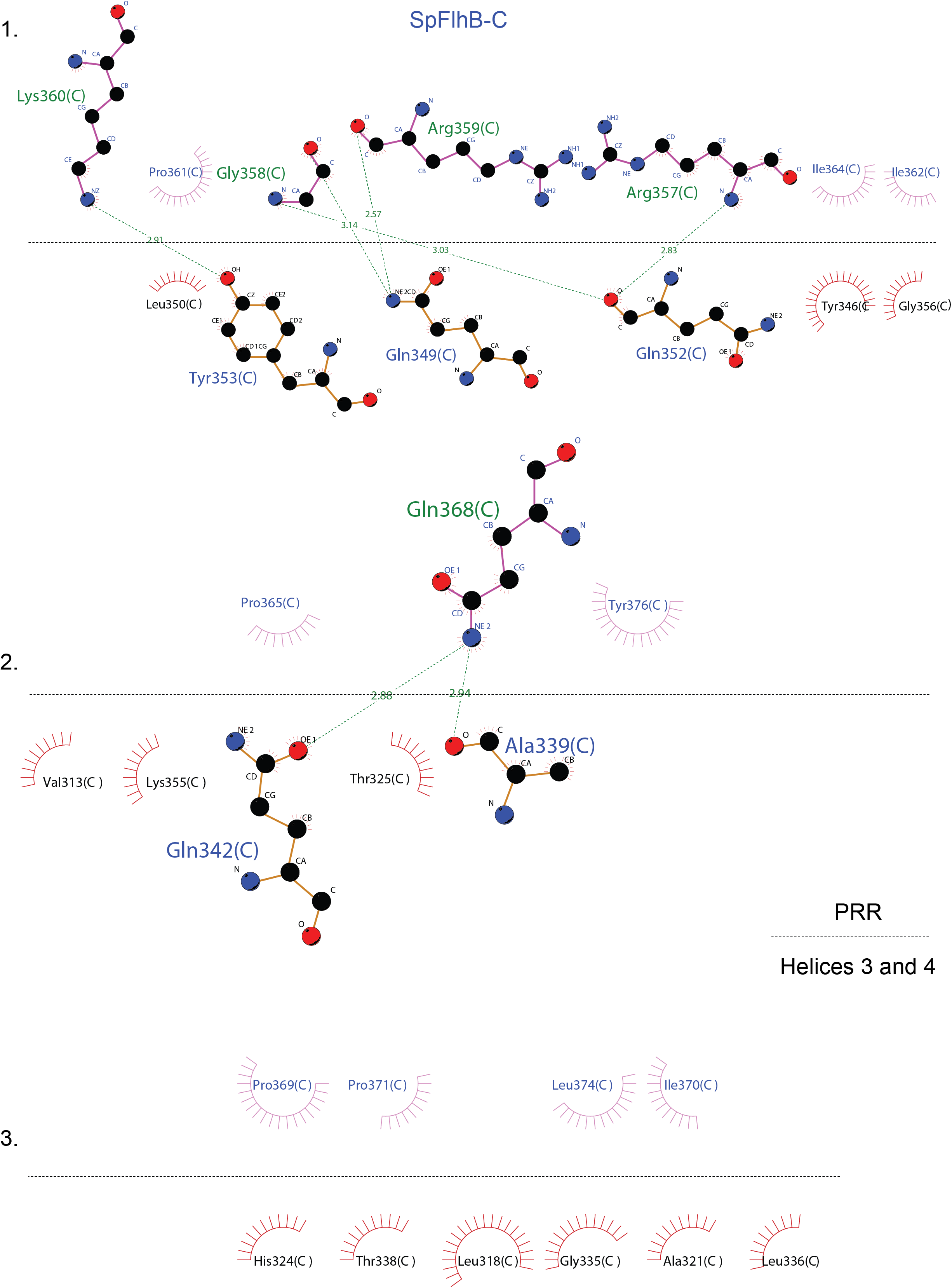
**

**Supplementary Fig. S1. LigPlot DIMPLOT data** **output**. Residues directly interacting with one another are fully depicted, with interacting atoms indicated. Residues forming hydrophobic contacts to only are depicted by name and residue number. The residues on the top half of panels 1, 2 and 3 are those contained in the PRR (357-376); those in the bottom half of all panels, under the dotted line, are residues from helices 3 and 4 of FlhB-C (residues 312-356). Data originally obtained as single horizontal image but rearranged to fit into a vertical layout.


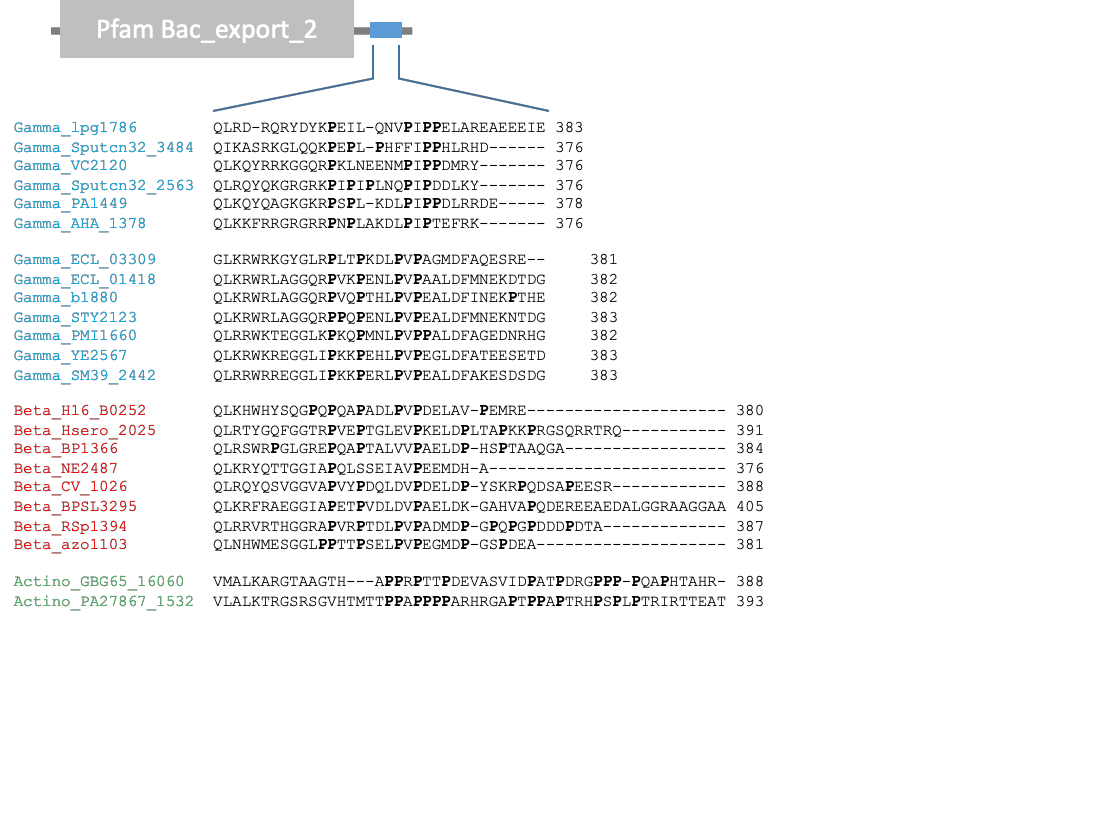


**Supplementary Fig. S2.** Examples of PRRs within the C-terminal tail of FlhB. Examples of PRR for the γ-proteo, β-proteo and Actinobacteria are shown with grouping and order of proteobacterial motifs according to figure 6b.

**Supplementary References**

1. Miller VL & Mekalanos JJ (1988) A novel suicide vector and its use in construction of insertion mutations: osmoregulation of outer membrane proteins and virulence determinants in Vibrio cholerae requires toxR. *J Bacteriol* 170(6):2575-2583.

2. Rossmann FM*, et al.* (2019) The GGDEF Domain of the Phosphodiesterase PdeB in Shewanella putrefaciens Mediates Recruitment by the Polar Landmark Protein HubP. *J Bacteriol* 201(7).

3. Kuhn MJ, Schmidt FK, Eckhardt B, & Thormann KM (2017) Bacteria exploit a polymorphic instability of the flagellar filament to escape from traps. *Proc Natl Acad Sci U S A* 114(24):6340-6345.

4. Lassak J, Henche AL, Binnenkade L, & Thormann KM (2010) ArcS, the cognate sensor kinase in an atypical Arc system of Shewanella oneidensis MR-1. *Appl Environ Microbiol* 76(10):3263-3274.
